# Supplementary material for: Content-rich biological network constructed by mining PubMed abstracts
Source: BMC Bioinformatics. 2004 Oct 8;5:147. doi: 10.1186/1471-2105-5-147 (PMC528731; doi:10.1186/1471-2105-5-147)
Supplement: Additional File 5 — The original Chilibot query results of the term "long-term potentiation (LTP)" and 22 other terms, limiting the latest references analyzed to the years 1990, 1995, 2000, and 2004. [file 1471-2105-5-147-S5.bz2 › chilibotAdditionalFile5/ltp1995/html/ERK_PI-3K.html]

 


 **ERK** and **PI-3K** 
  
Found 2 abstracts in PubMed,  **2 abstracts were retrieved and analyzed**.  


---

 Search Google  |
 PDF files only 
|  EDU domain only 

---

**Interactive relationship** (e.g. stimulation, inhibition, etc)

- Insulin as a key factor in the process of the adipocyte differentiation activated PI 3 kinase  [ **PI-3K** ] , Ras protein, and mitogen activated protein kinase MAP kinase,  **ERK**  in 3T3 L1 fibroblasts.  Ref: 7755636 Biochem Biophys Res Commun, 1995

- :-)

  **Parallel relationship** (e.g. studied together, co-existance, homology, etc.)

  - and PI 3 kinase  [ **PI-3K** ]  and  **ERK**  activation.  Ref: 7646892 Neuron, 1995
